# Supplementary material for: Modeling glioblastoma heterogeneity as a dynamic network of cell states
Source: Mol Syst Biol. 2021 Sep 16;17(9):e10105. doi: 10.15252/msb.202010105 (PMC8444284; doi:10.15252/msb.202010105)
Supplement: Supplementary file 5 — Source Data for Figure 3 [file MSB-17-e10105-s001.zip › Figure3A_sourcedata/GSEA_3065/hallmarks_state2.GseaPreranked.1623416269049/HALLMARK_APOPTOSIS.html]

Details for gene set HALLMARK\_APOPTOSIS[GSEA]

|  || Dataset | state2 |
| Phenotype | NoPhenotypeAvailable |
| Upregulated in class | na\_neg |
| GeneSet | HALLMARK\_APOPTOSIS |
| Enrichment Score (ES) | -0.3828419 |
| Normalized Enrichment Score (NES) | -1.4831848 |
| Nominal p-value | 0.008097166 |
| FDR q-value | 0.027462624 |
| FWER p-Value | 0.198 |
Table: GSEA Results Summary

  

Fig 1: Enrichment plot: HALLMARK\_APOPTOSIS      
 Profile of the Running ES Score & Positions of GeneSet Members on the Rank Ordered List

  

| PROBE | GENE SYMBOL | GENE\_TITLE | RANK IN GENE LIST | RANK METRIC SCORE | RUNNING ES | CORE ENRICHMENT || 1 | HMGB2 |  |  | 4 | 0.823 | 0.0684 | No |
| 2 | TOP2A |  |  | 15 | 0.596 | 0.1171 | No |
| 3 | BRCA1 |  |  | 92 | 0.381 | 0.1408 | No |
| 4 | BTG3 |  |  | 173 | 0.295 | 0.1569 | No |
| 5 | CCND2 |  |  | 227 | 0.263 | 0.1732 | No |
| 6 | CDK2 |  |  | 382 | 0.208 | 0.1742 | No |
| 7 | GPX4 |  |  | 425 | 0.194 | 0.1859 | No |
| 8 | DNAJA1 |  |  | 513 | 0.178 | 0.1914 | No |
| 9 | CDC25B |  |  | 519 | 0.178 | 0.2058 | No |
| 10 | CCND1 |  |  | 840 | 0.134 | 0.1827 | No |
| 11 | SOD1 |  |  | 1028 | 0.117 | 0.1724 | No |
| 12 | VDAC2 |  |  | 1083 | 0.112 | 0.1761 | No |
| 13 | BID |  |  | 1508 | 0.086 | 0.1378 | No |
| 14 | FEZ1 |  |  | 1543 | 0.084 | 0.1412 | No |
| 15 | WEE1 |  |  | 1617 | 0.081 | 0.1402 | No |
| 16 | RELA |  |  | 1694 | 0.077 | 0.1385 | No |
| 17 | ROCK1 |  |  | 1729 | 0.076 | 0.1412 | No |
| 18 | GSR |  |  | 1739 | 0.075 | 0.1465 | No |
| 19 | ETF1 |  |  | 1749 | 0.075 | 0.1518 | No |
| 20 | GADD45A |  |  | 1791 | 0.073 | 0.1535 | No |
| 21 | JUN |  |  | 1805 | 0.072 | 0.1581 | No |
| 22 | CASP3 |  |  | 2028 | 0.064 | 0.1397 | No |
| 23 | PEA15 |  |  | 2161 | 0.059 | 0.1304 | No |
| 24 | RHOB |  |  | 2690 | 0.042 | 0.0774 | No |
| 25 | CASP2 |  |  | 2794 | 0.039 | 0.0696 | No |
| 26 | PPP3R1 |  |  | 2869 | 0.037 | 0.0648 | No |
| 27 | ENO2 |  |  | 3006 | 0.034 | 0.0530 | No |
| 28 | DNM1L |  |  | 3024 | 0.033 | 0.0540 | No |
| 29 | CASP7 |  |  | 3134 | 0.031 | 0.0449 | No |
| 30 | DFFA |  |  | 3267 | 0.028 | 0.0331 | No |
| 31 | CASP6 |  |  | 3357 | 0.027 | 0.0258 | No |
| 32 | SOD2 |  |  | 3366 | 0.026 | 0.0272 | No |
| 33 | ISG20 |  |  | 3567 | 0.023 | 0.0076 | No |
| 34 | HSPB1 |  |  | 3629 | 0.022 | 0.0029 | No |
| 35 | DAP |  |  | 3729 | 0.020 | -0.0060 | No |
| 36 | CDKN1B |  |  | 4019 | 0.015 | -0.0357 | No |
| 37 | RARA |  |  | 4228 | 0.011 | -0.0570 | No |
| 38 | BCL10 |  |  | 4493 | 0.007 | -0.0847 | No |
| 39 | BAX |  |  | 4506 | 0.007 | -0.0854 | No |
| 40 | PAK1 |  |  | 4543 | 0.007 | -0.0887 | No |
| 41 | DAP3 |  |  | 5044 | -0.001 | -0.1422 | No |
| 42 | TSPO |  |  | 5245 | -0.004 | -0.1633 | No |
| 43 | XIAP |  |  | 5371 | -0.006 | -0.1762 | No |
| 44 | BNIP3L |  |  | 5447 | -0.008 | -0.1836 | No |
| 45 | BCL2L11 |  |  | 5510 | -0.009 | -0.1895 | No |
| 46 | PMAIP1 |  |  | 5617 | -0.010 | -0.2000 | No |
| 47 | MADD |  |  | 5750 | -0.012 | -0.2131 | No |
| 48 | ANKH |  |  | 5757 | -0.012 | -0.2127 | No |
| 49 | PTK2 |  |  | 5931 | -0.015 | -0.2300 | No |
| 50 | CREBBP |  |  | 5967 | -0.016 | -0.2324 | No |
| 51 | EBP |  |  | 6195 | -0.020 | -0.2550 | No |
| 52 | DDIT3 |  |  | 6433 | -0.024 | -0.2784 | No |
| 53 | PPP2R5B |  |  | 6473 | -0.025 | -0.2805 | No |
| 54 | DPYD |  |  | 6623 | -0.028 | -0.2942 | No |
| 55 | ADD1 |  |  | 6688 | -0.029 | -0.2986 | No |
| 56 | ATF3 |  |  | 6737 | -0.030 | -0.3012 | No |
| 57 | RNASEL |  |  | 6942 | -0.035 | -0.3201 | No |
| 58 | BCL2L1 |  |  | 7048 | -0.038 | -0.3282 | No |
| 59 | NEDD9 |  |  | 7098 | -0.039 | -0.3302 | No |
| 60 | ANXA1 |  |  | 7265 | -0.044 | -0.3443 | No |
| 61 | TIMP3 |  |  | 7309 | -0.045 | -0.3451 | No |
| 62 | SLC20A1 |  |  | 7482 | -0.050 | -0.3594 | No |
| 63 | PSEN2 |  |  | 7558 | -0.052 | -0.3631 | No |
| 64 | IRF1 |  |  | 7563 | -0.052 | -0.3592 | No |
| 65 | PDCD4 |  |  | 7568 | -0.052 | -0.3552 | No |
| 66 | CTNNB1 |  |  | 7580 | -0.053 | -0.3520 | No |
| 67 | FDXR |  |  | 7726 | -0.057 | -0.3628 | No |
| 68 | SATB1 |  |  | 7741 | -0.058 | -0.3594 | No |
| 69 | PSEN1 |  |  | 7761 | -0.058 | -0.3566 | No |
| 70 | CYLD |  |  | 7873 | -0.062 | -0.3633 | No |
| 71 | SC5D |  |  | 7900 | -0.063 | -0.3608 | No |
| 72 | ERBB2 |  |  | 7902 | -0.063 | -0.3556 | No |
| 73 | TNFRSF12A |  |  | 7931 | -0.064 | -0.3533 | No |
| 74 | IFNGR1 |  |  | 7945 | -0.065 | -0.3493 | No |
| 75 | TAP1 |  |  | 7963 | -0.066 | -0.3456 | No |
| 76 | RHOT2 |  |  | 8124 | -0.073 | -0.3567 | No |
| 77 | MCL1 |  |  | 8200 | -0.077 | -0.3582 | No |
| 78 | ERBB3 |  |  | 8230 | -0.079 | -0.3548 | No |
| 79 | PPT1 |  |  | 8369 | -0.087 | -0.3623 | No |
| 80 | CFLAR |  |  | 8485 | -0.095 | -0.3667 | No |
| 81 | LMNA |  |  | 8636 | -0.106 | -0.3740 | Yes |
| 82 | IFITM3 |  |  | 8720 | -0.113 | -0.3734 | Yes |
| 83 | PLPPR4 |  |  | 8779 | -0.119 | -0.3696 | Yes |
| 84 | SQSTM1 |  |  | 8790 | -0.121 | -0.3606 | Yes |
| 85 | TGFB2 |  |  | 8921 | -0.137 | -0.3630 | Yes |
| 86 | SPTAN1 |  |  | 8923 | -0.138 | -0.3517 | Yes |
| 87 | TXNIP |  |  | 8987 | -0.147 | -0.3461 | Yes |
| 88 | BCAP31 |  |  | 9017 | -0.152 | -0.3365 | Yes |
| 89 | F2R |  |  | 9026 | -0.153 | -0.3246 | Yes |
| 90 | HMOX1 |  |  | 9084 | -0.163 | -0.3171 | Yes |
| 91 | IGF2R |  |  | 9107 | -0.167 | -0.3055 | Yes |
| 92 | CAV1 |  |  | 9130 | -0.172 | -0.2935 | Yes |
| 93 | PLAT |  |  | 9145 | -0.176 | -0.2802 | Yes |
| 94 | DNAJC3 |  |  | 9150 | -0.177 | -0.2658 | Yes |
| 95 | EMP1 |  |  | 9156 | -0.179 | -0.2514 | Yes |
| 96 | GSN |  |  | 9238 | -0.206 | -0.2429 | Yes |
| 97 | BTG2 |  |  | 9239 | -0.207 | -0.2256 | Yes |
| 98 | SAT1 |  |  | 9279 | -0.224 | -0.2111 | Yes |
| 99 | CDKN1A |  |  | 9301 | -0.230 | -0.1941 | Yes |
| 100 | MMP2 |  |  | 9307 | -0.235 | -0.1750 | Yes |
| 101 | LGALS3 |  |  | 9308 | -0.236 | -0.1552 | Yes |
| 102 | TIMP2 |  |  | 9379 | -0.316 | -0.1363 | Yes |
| 103 | TIMP1 |  |  | 9387 | -0.334 | -0.1091 | Yes |
| 104 | CD44 |  |  | 9407 | -0.397 | -0.0780 | Yes |
| 105 | APP |  |  | 9412 | -0.426 | -0.0428 | Yes |
| 106 | CLU |  |  | 9430 | -0.547 | 0.0011 | Yes |
Table: GSEA details [plain text format]

  

Fig 2: HALLMARK\_APOPTOSIS: Random ES distribution      
 Gene set null distribution of ES for **HALLMARK\_APOPTOSIS**
